# Neurological outcomes 1 year after COVID‐19 diagnosis: A prospective longitudinal cohort study

**DOI:** 10.1111/ene.15307

**Published:** 2022-03-23

**Authors:** Verena Rass, Ronny Beer, Alois Josef Schiefecker, Anna Lindner, Mario Kofler, Bogdan Andrei Ianosi, Philipp Mahlknecht, Beatrice Heim, Marina Peball, Federico Carbone, Victoria Limmert, Philipp Kindl, Lauma Putnina, Elena Fava, Sabina Sahanic, Thomas Sonnweber, Wolfgang N. Löscher, Julia V. Wanschitz, Laura Zamarian, Atbin Djamshidian, Ivan Tancevski, Günter Weiss, Rosa Bellmann‐Weiler, Stefan Kiechl, Klaus Seppi, Judith Loeffler‐Ragg, Bettina Pfausler, Raimund Helbok

**Affiliations:** ^1^ 27280 Department of Neurology Medical University of Innsbruck Innsbruck Austria; ^2^ 27280 Department of Internal Medicine II Medical University of Innsbruck Innsbruck Austria

**Keywords:** COVID‐19, long COVID, neurological manifestations, SARS‐CoV‐2

## Abstract

**Background and purpose:**

Neurological sequelae from coronavirus disease 2019 (COVID‐19) may persist after recovery from acute infection. Here, the aim was to describe the natural history of neurological manifestations over 1 year after COVID‐19.

**Methods:**

A prospective, multicentre, longitudinal cohort study in COVID‐19 survivors was performed. At a 3‐month and 1‐year follow‐up, patients were assessed for neurological impairments by a neurological examination and a standardized test battery including the assessment of hyposmia (16‐item Sniffin' Sticks test), cognitive deficits (Montreal Cognitive Assessment < 26) and mental health (Hospital Anxiety and Depression Scale and Post‐traumatic Stress Disorder Checklist 5).

**Results:**

Eighty‐one patients were evaluated 1 year after COVID‐19, out of which 76 (94%) patients completed a 3‐month and 1‐year follow‐up. Patients were 54 (47–64) years old and 59% were male. New and persistent neurological disorders were found in 15% (3 months) and 12% (10/81; 1 year). Symptoms at 1‐year follow‐up were reported by 48/81 (59%) patients, including fatigue (38%), concentration difficulties (25%), forgetfulness (25%), sleep disturbances (22%), myalgia (17%), limb weakness (17%), headache (16%), impaired sensation (16%) and hyposmia (15%). Neurological examination revealed findings in 52/81 (64%) patients without improvement over time (3 months, 61%, *p* = 0.230) including objective hyposmia (Sniffin' Sticks test <13; 51%). Cognitive deficits were apparent in 18%, whereas signs of depression, anxiety and post‐traumatic stress disorders were found in 6%, 29% and 10% respectively 1 year after infection. These mental and cognitive disorders had not improved after the 3‐month follow‐up (all *p* > 0.05).

**Conclusion:**

Our data indicate that a significant patient number still suffer from neurological sequelae including neuropsychiatric symptoms 1 year after COVID‐19 calling for interdisciplinary management of these patients.

## Graphical abstract

Out of 81 patients, 48 (59%) reported persistent symptoms that were not evident before COVID‐19 at the 1‐year follow‐up. New and persistent neurological disorders were found in 15% (3 months) and 12% (10/81; 1‐year). Cognitive deficits one year after COVID‐19 were apparent in 18%, whereas depression, anxiety, and post‐traumatic stress disorders were found in 6%, 29%, and 10% one year after infection, respectively. Our data indicate that a significant patient number still suffer from neurological sequelae including neuropsychiatric symptoms one year after COVID‐19.

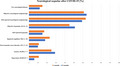

## INTRODUCTION

Neurological complications associated with acute coronavirus disease 2019 (COVID‐19) are well described [1]. Now, increasing evidence suggests that symptoms may persist even beyond 4–12 weeks after disease onset [2]. Apart from pulmonary and other organ manifestations, post‐COVID neurological symptoms have captured the interest of national societies and individual research groups [3, 4, 5]. Frequent neurological, cognitive and neuropsychiatric long‐term symptoms after COVID‐19 include but are not limited to headache, dizziness, difficulty in concentration, attention and memory, fatigue, insomnia, depression and anxiety [6]. In line with this, a meta‐analysis including 15 follow‐up studies of COVID‐19 patients evaluated between 14 and 110 days post‐infection, identified 55 long‐term effects of COVID‐19 with fatigue, headache, attention disorder, hair loss and dyspnoea as the most common symptoms [7].

Point‐prevalence studies commonly miss the dynamic evolution of symptoms and diseases over time and vary depending on the study design and the cohorts studied [5]. In a recent observational study including 1276 hospitalized patients, 68% reported at least one sequela 6 months after the acute infection with further improvement at the 1‐year follow‐up (49%) [8]. Fatigue (20%) and sleep disorders (17%) were the most prevalent neurological symptoms, and anxiety as well as depression affected 26% of patients at the 1‐year follow‐up [8].

Recently, a point‐prevalence study of neurological manifestations 3 months after acute disease in 135 COVID‐19 survivors was reported [4]. Accounting for pre‐existing neurological diseases, every sixth patient was diagnosed with a neurological disease, which was directly associated with COVID‐19 disease severity. Persistent hyposmia assessed with a licensed smelling test was common (45%), even in patients recovering from mild disease [4].

In the current study, the aim was to assess this prospective consecutive cohort again 12 months after disease onset, describing the natural history of neurological signs, symptoms and diseases as well as neurocognitive and neuropsychiatric complaints. As secondary objectives, the aim was to find predictors of persisting olfactory dysfunction, anxiety or depression, cognitive deficits and fatigue. It was hypothesized that neurological outcomes improved over time in a substantial number of patients.

## METHODS

### Study design, setting and participants

COVID‐19 patients prospectively included in this multicentre observational cohort study were recruited at three sites in Tyrol upon disease onset (Department of Internal Medicine II, Medical University of Innsbruck, the tertiary care centre Zams and the acute rehabilitation facility Münster). Recruited patients were diagnosed with COVID‐19 between March and June 2020. To this date, no variants of Sars‐CoV‐2 were reported. Details are given in our previous studies [4, 9]. Study inclusion criteria were (1) confirmed SARS‐CoV‐2 infection, (2) hospitalization or outpatient management with symptoms persisting for at least 6 weeks after initial COVID‐19 diagnosis and (3) age greater ≥18 years. Patients who died during the acute phase were not included in this study, and the intensive care unit (ICU) mortality rate was 19% during the acute phase of the study as reported elsewhere [10]. Only some of these patients were evaluated for neurological complications during the acute disease.

### Standard protocol approvals, registrations and patient consents

The local ethics committee approved the conduct of the study (Medical University of Innsbruck, EK No. 1103/2020) and the study was registered at ClinicalTrials.gov (NCT05025839). Written informed consent was obtained from all patients according to local regulations.

### Study procedures and data collection

Detailed data collection procedures at the 3‐month follow‐up are described elsewhere [4, 9]. In brief, all patients received a cardiopulmonary 3‐month follow‐up including clinical examination, standard laboratory examinations and a low‐dose computed tomography (CT) scan of the chest. The patients also underwent a 3‐month neurological examination consisting of a structured interview, olfactory testing, cognitive testing and assessment of anxiety, depression as well as quality of life.

At the 1‐year follow‐up, patients underwent the same test battery. A detailed interview was performed to assess the frequency of self‐perceived sequelae including 16 symptoms and their evolution over time (any time, ≥4 weeks, ≥3 months, ongoing). The standardized neurological examination to assess neurological signs or diseases was done by neurological consultants or registrars under the direct supervision of a consultant. To assess the olfactory function, the 16‐item Sniffin' Sticks identification test (SS‐16; Burghart Medizintechnik) was used: the nasal chemosensory performance was evaluated using pen‐like odour dispensing devices for odour identification of 16 common odorants (multiple forced‐choice from four verbal items per test odorant) [11]. Hyposmia and anosmia were determined using cut‐off levels of <13 and <9 respectively, as per manufacturer criteria.

Cognitive deficits were screened for using the Montreal Cognitive Assessment (MoCA), and impairment was classified in patients scoring below 26/30 points [12].

Post‐traumatic stress, depression and anxiety were captured with the Post‐traumatic Stress Disorder Checklist 5 (PCL‐5) [13] and the Hospital Anxiety and Depression Scale (HADS) [14]. The PCL‐5 captures 20 symptoms each with 0 to 4 points resulting in a total score of 0 to 80. Higher sums are indicative of post‐traumatic stress disorder (PTSD). Scores >32 indicate clinically relevant PTSD. The HADS measures levels of anxiety and depression during the last week. It consists of an anxiety (HADS‐A) and depression (HADS‐D) subscale each testing seven items scoring from 0 to 3. Scores range from 0 to 21 in each subscale. Lower scores are linked to less severe anxiety‐ and depression‐related symptoms. Scores >7 suggest mild symptom burden, >10 a clinically meaningful anxiety disorder or depression [15].

Fatigue was assessed by self‐report (yes/no) and by use of the fatigue assessment scale (FAS) and the fatigue severity scale (FSS). The FAS is a 10‐item questionnaire to assess mental (five questions) and physical (five questions) fatigue with each question scoring from 1 to 5. By summing up the points on all questions, the total score ranges from 10 to 50. A total FAS score >21 indicates fatigue [16]. The FSS contains nine questions that rate the severity of fatigue symptoms based on a 7‐point Likert scale [17]. The total FSS score represents the mean sum of the nine items, therefore ranging between 1 and 7. A mean FSS score ≥5 indicates clinically significant fatigue [18].

Functional outcome was assessed with use of the Glasgow Outcome Scale Extended (GOSE) and the modified Rankin Scale (mRS) score.

Disease severity groups were defined according to the required invasiveness during the acute disease: (1) non‐hospitalized (mild) patients, (2) hospitalized (moderate) patients not requiring ICU admission and (3) COVID‐19 patients admitted to the ICU (severe).

### Statistical analysis

Categorical variables are given in counts and percentages and continuous variables are summarized using univariate statistical measures including medians and interquartile ranges or means and standard deviations. The McNemar test or paired *t* test was used to check for changes between the 3‐month and 1‐year follow‐up. All results are given for the different disease severity groups. Based on data distribution (Kolmogorov–Smirnov test and Shapiro–Wilk test) parametric or non‐parametric tests were applied. The chi‐squared or Kruskal–Wallis test was used to assess for differences across severity grades (Tables S1–S3).

To assess independent predictors of objective hyposmia (SS‐16 <13), impaired cognition (MoCA <26), signs of anxiety or depression, or fatigue (FAS >21) at the 1‐year follow‐up, multivariable logistic regression analysis was employed. For model selection, a multi‐step approach was used. Out of a total of 64 variables including demographics, premedical history, symptoms/treatments during acute disease, laboratory parameters, neurological/neuropsychiatric signs or diseases, cardiopulmonary symptoms and CT abnormalities obtained at the 3‐month follow‐up, variables were selected to build our full models. The initial selection was based on variable importance using the random forest package for R (version 4.6‐14). Thereby, the first 10 variables which produced the most decrease in model accuracy and the first 10 that produced a decrease of Gini impurity were pre‐selected. Then, a stepwise forward and backward selection based on the Akaike information criterion (AIC) of generalized linear models was used and collinear factors based on the variance inflation factor were excluded. For this analysis, the R package MASS (version 7.3‐53.1) was used with the StepAIC function and adjusted odds ratios were calculated with 95% confidence intervals. Missing data were indicated for descriptive analysis and imputed using multiple imputations based on random forests with the missForest package for R (version 1.4). The vast majority of imputed variables were categorical and had less than 15% missing values.

A two‐sided *p* value <0.05 was considered statistically significant. All analyses were performed with SPSS (IBM SPSS Statistics, Version 24.0) and R.

## RESULTS

In total, 76 of 135 patients who presented at the 3‐month follow‐up completed the 1‐year follow‐up. In addition five patients who were evaluated at 1 year only were recruited, resulting in a total of 81 patients who completed in‐person follow‐up 1 year after COVID‐19 diagnosis (median 416 days, interquartile range 401–437). The median age was 54 (47–64) years, and the majority were male patients (59%). All initial severity grades were included, reflected by the necessity of ICU care (*n* = 20/81, 25%), the requirement for admission to the normal hospital ward (*n* = 35/81, 43%) and outpatients (*n* = 26/81, 32%) (Table 1). There was no difference in demographics and disease severity in patients completing consecutive follow‐ups (3 months and 1 year) and those only seen at 3 months (disease severity, *p* = 0.116; age, *p* = 0.103; sex, *p* = 0.860).

**TABLE 1:** Demographics, comorbidities and therapy in 81 patients according to COVID‐19 severity

	All *N* = 81	Severe disease requiring ICU admission *n* = 20 (25%)	Moderate severity (hospitalization, non‐ICU) *n* = 35 (43%)	Mild severity (outpatient) *n* = 26 (32%)	*p* value*
Age (years)	54 (47–64)	54 (49–63)	63 (54–73)	46 (36–54)	<0.001
Sex (female)	33 (41)	6 (30)	10 (29)	17 (65)	0.008
Body mass index	26 (24–29)	26 (24–31)	27 (25–30)	25 (21–29)	0.281
Current smoking	3 (4)	0 (0)	2 (6)	1 (4)	0.561
Ex‐smoking	33 (41)	7 (35)	20 (57)	6 (24)	0.030
Pack years	8 ± 14	7 ± 15	13 ± 15	2 ± 6	0.008
Premedical history					
Cardiovascular disease	32 (40)	12 (60)	19 (54)	1 (4)	<0.001
Arterial hypertension	26 (32)	10 (50)	15 (43)	1 (4)	0.001
Pulmonary disease	16 (20)	4 (20)	7 (20)	5 (19)	0.997
Endocrinological disease	31 (38)	8 (40)	18 (51)	5 (19)	0.037
Hypercholesterolaemia	16 (20)	3 (15)	12 (34)	1 (4)	0.011
Diabetes mellitus II	12 (15)	5 (25)	6 (17)	1 (4)	0.118
Chronic kidney disease	4 (5)	2 (10)	2 (6)	0 (0)	0.288
Chronic liver disease	4 (5)	2 (10)	2 (6)	0 (0)	0.288
Malignancy	8 (10)	0 (0)	7 (20)	1 (4)	0.026
Immunological deficiency	3 (4)	2 (10)	0 (0)	1 (4)	0.168
Pre‐existing neurological diseases					
None	62 (77)	17 (85)	24 (69)	21 (81)	0.318
Stroke	3 (4)	1 (5)	2 (6)	0 (0)	0.457
Parkinsonism	0 (0)	0 (0)	0 (0)	0 (0)	–
Multiple sclerosis	0 (0)	0 (0)	0 (0)	0 (0)	–
Motor neuron disease	0 (0)	0 (0)	0 (0)	0 (0)	–
Neuropathy	3 (4)	0 (0)	3 (9)	0 (0)	0.129
Traumatic brain injury	2 (3)	1 (5)	1 (3)	0 (0)	0.546
Restless legs syndrome	1 (1)	0 (0)	0 (0)	1 (4)	0.328
Essential tremor	0 (0)	0 (0)	0 (0)	0 (0)	–
Migraine	3 (4)	0 (0)	1 (3)	2 (8)	0.380
Neuromuscular disease	0 (0)	0 (0)	0 (0)	0 (0)	–
Epilepsy	0 (0)	0 (0)	0 (0)	0 (0)	–
Poliomyelitis	1 (1)	1 (5)	0 (0)	0 (0)	0.328
Treatment and hospital course					
Oxygen requirement	37 (46)	20 (100)	18 (51)	0 (0)	<0.001
Mechanical ventilation	18 (23)	19 (95)	0 (0)	0 (0)	<0.001
Steroid treatment	18 (23)	7 (37)	5 (14)	1 (4)	0.011
Length of hospital stay (days)	8 (0–18)	31 (24–49)	9 (6–12)	0 (0–0)	<0.001

Note

Data are given as median (interquartile range), mean ± SD and counts (%).

Abbreviation: ICU, intensive care unit.

* Chi‐squared or Kruskal–Wallis tests were used to assess for differences across severity grades (severe, moderate, mild). A *p* value <0.05 signifies a significantly different data distribution across severity groups.

### Neurological diseases

**FIGURE 1:**
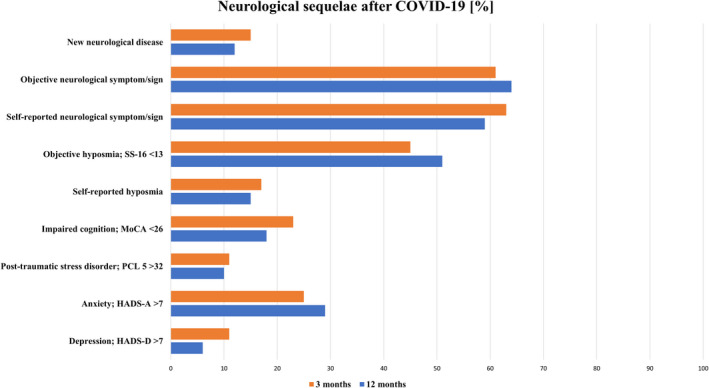
Neurological sequelae 3 months and 1 year after being diagnosed with COVID‐19. SS‐16, 16‐item Sniffin' Sticks identification test; MoCA, Montreal Cognitive Assessment; PCL‐5, Post‐traumatic Stress Disorder Checklist; HADS, Hospital Anxiety and Depression Scale [Colour figure can be viewed at wileyonlinelibrary.com]

At least one neurological disease not diagnosed prior to COVID‐19 was found in 10/81 patients (12%) at the 1‐year follow‐up: neuropathy/myopathy in eight patients (distal symmetric axonal neuropathy [*n* = 3], small fibre neuropathy [*n* = 1], critical illness polyneuropathy and myopathy [CIP/CIM] [*n* = 1], compression neuropathy [*n* = 3]), mild encephalopathy (*n* = 1) and newly diagnosed Parkinson's disease in two patients (Tables 2 and S1). Although a statistical improvement over time was not found (15% at 3‐month follow‐up vs. 12% at 1‐year follow‐up; *p* = 0.453; Figure 1), four patients recovered from CIP/CIM, two from mild encephalopathy, and one from orthostatic hypotension with vasovagal syncope between the 3‐month and 1‐year follow‐up.

**TABLE 2:** Neurological diseases 3 months and 1 year after COVID‐19 diagnosis

	3‐month follow‐up *n* = 135	1‐year follow‐up *n* = 81	*p* value* *n* = 76
Any neurological disease not diagnosed prior to COVID‐19	20 (15)	10 (12)	0.453
Neuropathy/myopathy	16 (12)	8 (9)	
CIP/CIM	8 (6)	1 (1)	0.125
Symmetric axonal distal neuropathy	7 (5)	3 (4)	0.500
Small fibre neuropathy	0 (0)	1 (1)***	–
Compression neuropathy	3 (2)	3 (4)	
Guillain–Barré syndrome	1 (1)**	0 (0)	–
Parkinsonism	1 (1)**	2 (2)	–
Cerebellar ataxia	0 (0)	0 (0)	–
Mild encephalopathy	2 (2)	1 (1)***	–
Ischaemic stroke	1 (1)**	0 (0)	–
Haemorrhagic stroke	0 (0)	0 (0)	–
Orthostatic hypotension	1 (1)	0 (0)	1.000
Seizures	0 (0)	0 (0)	–
Myelopathy	0 (0)	0 (0)	–

Abbreviation: CIP/CIM, critical illness polyneuropathy and myopathy.

* The McNemar test was used to evaluate changes between the 3‐month and 1‐year follow‐up

** Not at the 1‐year follow‐up

*** Not at the 3‐month follow‐up.

### Self‐reported and objective neurological signs and symptoms

**FIGURE 2:**
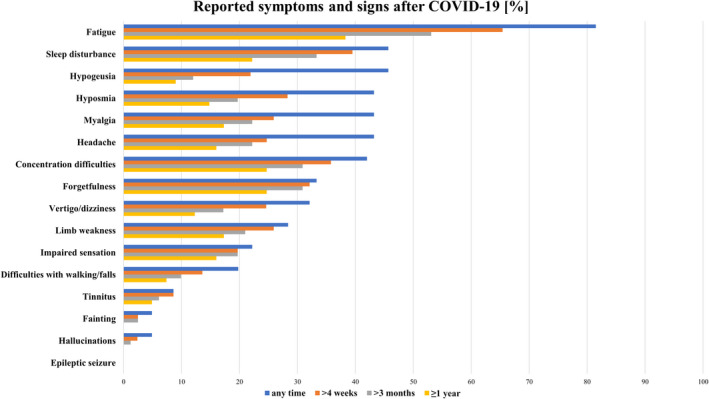
Self‐reported symptoms quantified based on the duration (any time, >4 weeks, >3 months, ≥1 year) [Colour figure can be viewed at wileyonlinelibrary.com]

The majority (48/81, 59%) reported at least one persisting neurological symptom 1 year after COVID‐19 diagnosis: these included fatigue (38%), concentration difficulties (25%), forgetfulness (25%), sleep disturbance (22%), myalgia (17%), limb weakness (17%), headache (16%), impaired sensation (16%), hyposmia (15%), vertigo/dizziness (12%), hypogeusia (9%), difficulties with walking/falls (7%) and tinnitus (5%). In Figure 2 neurological symptoms are quantified based on their duration (any time, >4 weeks, >3 months, ≥1 year). Internal reliability of self‐reported measures was high (Cronbach's alpha 0.812).

Objective neurological examination at 1‐year follow‐up revealed abnormalities in 52/81 (64%) patients with a predominance in hospitalized patients (severe, 80%; intermediate, 74%; mild, 39%; *p* = 0.004; Table S2). These included objective hyposmia (51%), abnormal reflex status (20%) and positive frontal release signs (10%; Table 3). When excluding patients with objective hyposmia as a unique symptom (SS‐16 <13), the neurological examination was not normal in 31/81 (38%) patients. Although some patients showed an improvement in the neurological examination (*n* = 18, 24%), there was no significant difference between 3‐month and 1‐year follow‐up (*p* = 0.230, Figure 1). A new neurological abnormality was documented in 17 patients (21%), although in only two patients (2%) it was probably attributed to COVID‐19; these symptoms included rigidity and reduced proprioception (hyposmia excluded).

**TABLE 3:** Neurological signs and symptoms 3 months and 1 year after COVID‐19 diagnosis

	3‐month follow‐up *n* = 135	1‐year follow‐up *n* = 81	*p* value* *n* = 76
Any neurological sign or symptom	82 (61)	52 (64)	0.230
Hyposmia/anosmia, SS‐16 <13	57 (45)	41 (51)	0.265
SS‐16 12–9 items correct	48 (38)	33 (41)	–
SS‐16 ≤8 items correct	9 (7)	8 (10)	–
Hyposmia/anosmia, SS‐16 <12	37 (29)	25 (31)	0.774
SS‐16	13 (11–14)	12 (11–14)	0.776
Neck stiffness	0 (0)	0 (0)	–
Decreased consciousness	0 (0)	0 (0)	–
Dysarthria	3 (2)	0 (0)	–
Aphasia	0 (0)	0 (0)	–
Positive frontal release signs	20 (15)	8 (10)	1.000
Anisocoria	0 (0)	0 (0)	–
Oculomotor nerve palsy	0 (0)	0 (0)	–
Facial palsy	0 (0)	0 (0)	–
Dysphagia	0 (0)	0 (0)	–
Bradykinesia	7 (5)	5 (6)	1.000
Dystonia	0 (0)	0 (0)	–
Chorea	0 (0)	0 (0)	–
Myoclonus/jerks	0 (0)	0 (0)	–
Asterixis	0 (0)	0 (0)	–
Dysmetria	2 (2)	2 (2)	1.000
Tremors	13 (10)	2 (2)	0.125
Abnormal muscle tone	6 (4)	5 (6)	0.453
Rigidity	3 (2)	5 (6)	–
Spasticity	2 (2)	0 (0)	–
Decreased muscle tone	1 (1)	0 (0)	–
Muscle atrophy	9 (7)	3 (4)	1.00
Reduced proprioception	20 (15)	14 (17)	0.581
Abnormal reflex status	31 (23)	16 (20)	0.508
Paresis	7 (5)	4 (5)	1.000
Babinski sign	2 (2)	1 (1)	1.000
Gait abnormality	7 (5)	6 (7)	0.688

Note

Data are given as count (%) and median (interquartile range).

Abbreviation: SS‐16, 16‐item Sniffin' Sticks test.

* The McNemar test or paired *t* test were used to evaluate changes between the 3‐month and 1‐year follow‐up.

### Self‐reported and objective hyposmia

Self‐reported hyposmia was uncommon (12/81, 15%) compared to objective hyposmia (SS‐16 <13) 1 year after COVID‐19 (51%). Overall, objective hyposmia did not change over time (at 3 months 45%, *p* = 0.265). However, individual patient data revealed worsening in the detection of odours in 25%, whilst 18% showed improved odour identification compared to their 3‐month follow‐up. When using a more liberal cut‐off (SS‐16 <12), 31% scored positive for hyposmia 1 year after COVID‐19.

### Cognition, mental health and functional outcome

Cognitive impairment was evident in 18% after 1 year compared to 23% at the 3‐month follow‐up (*p* = 1.00; Table 4) with more hospitalized patients being affected (severe, 24%; intermediate, 30%; mild, 0%; *p* = 0.001; Table S3). Signs of anxiety or depression were evident in 29% (3 months, 25%; *p* = 1.00) and 6% (3 months, 11%; *p* = 0.375). Ten per cent had signs of PTSD at the 1‐year follow‐up (3 months, 11%; *p* = 0.625).

**TABLE 4:** Mental health, cognition and functional outcome 3 months and 1 year after COVID‐19 diagnosis

	3‐month follow‐up *n* = 135	1‐year follow‐up *n* = 81	*p* value*	Missing values at 1‐year follow‐up**
Mental health				
Post‐traumatic stress disorder (PCL‐5 >32)	11 (11)	5 (10)	0.625	31
Depression (HADS‐D)	11 (11)	3 (6)	0.375	29
Depression (HADS‐D) >7	8 (8)	1 (2)	–	29
Depression (HADS‐D) >10	3 (3)	2 (4)	–	29
Anxiety (HADS‐A)	24 (25)	15 (29)	1.00	29
Anxiety (HADS‐A) >7	17 (17)	9 (17)	–	29
Anxiety (HADS‐A) >10	7 (7)	6 (12)	–	29
Fatigue measures				
FAS	–	22 (18–26)	–	13
FAS >21	–	34 (50)	–	13
FSS	–	3 (2–5)	–	24
FSS ≥5	–	11 (19)	–	24
Cognition				
MoCA (<26)	29 (23)	14 (18)	1.000	5
MoCA	28 (26–29)	28 (26–29)	0.184	5
Functional outcome				
GOSE	8 (7–8)	8 (7–8)	0.129	0
mRS	1 (0–1)	0 (0–1)	0.096	0

Notes

Data are given as median (interquartile range) and counts (%).

Anxiety and depression (HADS‐D) were scored as slightly increased when >7 and increased when >10.

Abbreviations: FAS, fatigue assessment scale; FSS, fatigue severity scale; GOSE, Glasgow Outcome Scale Extended; HADS, Hospital Anxiety and Depression Scale; MoCA, Montreal Cognitive Assessment; mRS, modified Rankin Scale score; PCL‐5, Post‐traumatic Stress Disorder Checklist.

* McNemar or paired *t* tests were used to assess for differences across the 3‐month and 1‐year follow‐up.

** For missing values at 3‐month follow‐up see [5].

Ongoing fatigue was self‐reported by 38% with similar frequencies across severity grades (severe, 45%; intermediate, 34%; mild, 39%; *p* = 0.734). With the use of the FAS, 50% scored >21 indicative of fatigue, whilst in the FSS 19% qualified for clinically significant fatigue.

Comparable to the 3‐month follow‐up, functional outcome was good with a median mRS of 0 (0–1) or GOSE of 8 (7–8) 1 year after COVID‐19 diagnosis.

### Factors predicting hyposmia, impaired cognition, mental health and fatigue

**FIGURE 3:**
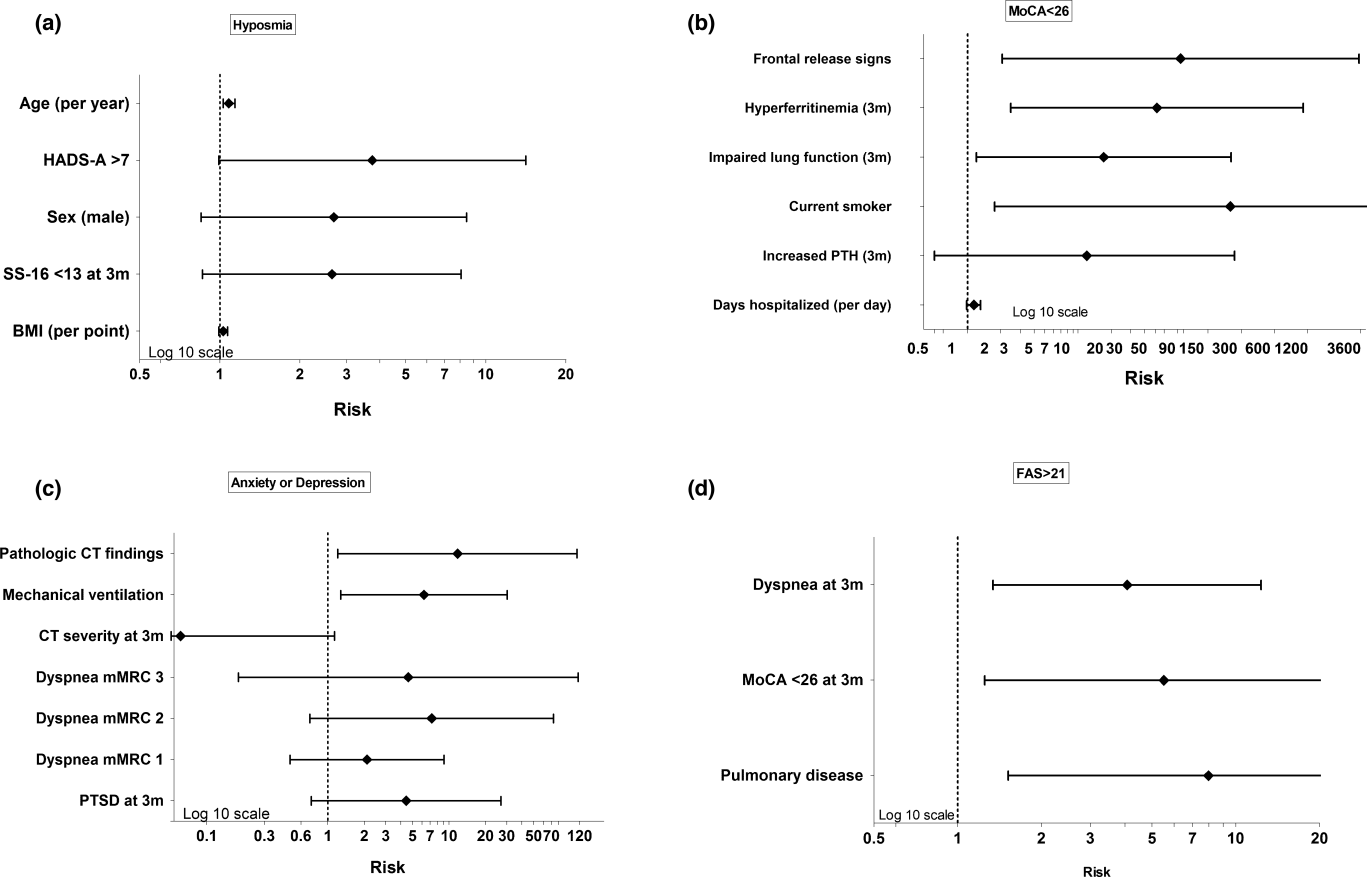
Factors associated with (a) objective hyposmia (SS‐16 <13), (b) impaired cognition, MoCA <26, (c) signs of anxiety or depression, and (d) fatigue, FAS >21, with calculated adjusted odds ratios based on logistic regression with the 95% confidence intervals. MoCA, Montreal Cognitive Assessment; FAS, Fatigue Assessment Scale; SS‐16, 16‐item Sniffin' Sticks test

The only independent associated factor with objective hyposmia (SS‐16 <13) at the 1‐year follow‐up was increasing age (adjusted OR per year [adjOR] 1.08, 95% confidence interval [CI] 1.01–1.15, *p* = 0.021; Figure 3a).

Multivariable analysis revealed positive frontal release signs at 3 months (adjOR 84.45, 95% CI 2.05–3477.97, *p* = 0.019), hyperferritinemia at 3 months (adjOR 51.64, 95% CI 2.45–1087.74, *p* = 0.011), impaired lung function at 3 months (adjOR 17, 95% CI 1.2–241.76, *p* = 0.036) and current smoking (adjOR 238.43, 95% CI 1.75–32,554.29, *p* = 0.029) being associated with MoCA <26 (Figure 3b).

Mechanical ventilation during the acute phase (adjOR 6.13, 95% CI 1.26–29.87, *p* = 0.025) and pathological findings on the chest CT scan obtained at 3 months (adjOR 11.6, 95% CI 1.19–112.83, *p* = 0.035) were associated with signs of anxiety or depression 1 year after COVID‐19 diagnosis (Figure 3c).

Factors associated with FAS >21 in multivariable analysis included pulmonary disease in premedical history (adjOR 7.97, 95% CI 1.52–41.92, *p* = 0.014), persistent dyspnoea at the 3‐month follow‐up (adjOR 4.07, 95% CI 1.34–12.33, *p* = 0.013) and cognitive impairment at the 3‐month follow‐up (adjOR 75.51, 95% CI 1.25–24.37, *p* = 0.024; Figure 3d).

## DISCUSSION

In this prospective longitudinal observational study, the natural history of neurological recovery from COVID‐19 up to 1 year after diagnosis is described. The focus was on new and persistent neurological symptoms and diseases as well as mental health/fatigue measures in a mixed population of outpatients and inpatients. A new onset mostly mild neurological disease within 12 months post‐COVID was found in 12% of the cohort. The most common self‐reported ongoing symptom was fatigue (38%) followed by concentration difficulties (25%), forgetfulness (25%) and sleep disturbance (22%). Objective and relevant neurological signs are described in 64% of patients with objective hyposmia (51%) being the most prevalent symptom. Impaired cognition (18%), signs of anxiety (29%) and depression (6%) were still present in a considerable number of patients. In contrast to other reports, a significant improvement over time was not found in all studied domains.

It is now well accepted that COVID‐19 may impact humans’ health beyond acute infection [2, 19]. Besides pulmonary manifestations and other organ dysfunctions, neuropsychiatric symptoms and signs gain attention as long‐term effects of COVID‐19 with fatigue, headache and attention disorders being common representations [7].

Fatigue was a prominent symptom reported by 38% after 1 year. Using the FAS, 50% scored positive for fatigue, and 19% qualified for disabling fatigue in the FSS. Despite this discrepancy dependent on the scale used, our data are comparable with a point‐prevalence rate of 20% recently reported by Huang et al. 1 year after COVID‐19 with the use of a single question item [8]. Post‐infection fatigue syndromes are well described after other viral and bacterial infections [20, 21] and symptoms also overlap with patients suspected of myalgic encephalomyelitis/chronic fatigue syndrome, where patients report infectious diseases before symptom onset. Underlying causes of fatigue in COVID‐19 patients may therefore not be specific for SARS‐CoV‐2 infection [20]. Sustained organ dysfunction involving the heart, lung and kidney are postulated to trigger chronic fatigue [22]. Accordingly, it was found that dyspnoea at the 3‐month follow‐up and pulmonary disease in premedical history were associated with persistent fatigue at 1 year. Another hypothesis is based on an ongoing low‐grade (neuro)‐inflammation after acute symptomatic COVID‐19. Neuroinflammation may be triggered by the virus itself [23] or by systemic inflammation activating the innate immune system in the brain and by up‐regulation of various cytokines [24]. This can impact on neurotransmitter synthesis specifically tryptophan degradation and subsequently limited serotonin synthesis [25]. In our cohort, systemic inflammatory markers such as C‐reactive protein or interleukin‐6 being associated with fatigue could not be confirmed; however, local–regional low‐grade immune activation in the central nervous system may not be reflected by alterations of circulating markers of inflammation. Interestingly, impaired cognition was associated with fatigue and may refer to the common symptom of mild cognitive impairment in patients with fatigue [19].

Cognitive impairment, objectively assessed using the MoCA, was prevalent in 23% after 3 months and 18% after 1 year, whilst even more patients (25%) reported persistent forgetfulness and concentration difficulties 1 year after acute COVID‐19. Several authors postulate that systemic infection and neuroinflammation may promote cognitive decline or even neurodegeneration after COVID‐19 [19, 26, 27]. This hypothesis is also supported by the association between inflammatory markers (e.g., procalcitonin and interleukin‐6) with hippocampal atrophy in patients with severe sepsis [28]. Moreover, moderate to severe cognitive impairment was observed in survivors of severe sepsis even 8 years after acute disease [29]. In our cohort, persistently elevated ferritin levels 3 months after acute COVID‐19 were associated with impaired cognition. Ferritin is a well‐known marker of inflammation [30], and hyperferritinemia as a manifestation of a hyperinflammatory state has been described in COVID‐19 patients [31] being associated with a more severe course of the infection [32]. Accordingly, hyperferritinemia was associated with persisting lung pathologies 60 days post COVID‐19 and correlated with increased cytokine mRNA expression in peripheral blood cells of patients [33]. Along this line, impaired lung function 3 months after acute COVID‐19 was identified as a risk factor for cognitive deficits at 1‐year follow‐up, which would also point to inflammation and radical mediated neurological damage and hormone dysfunction. Patients with SARS‐CoV‐2 lung injury are further at risk of hypoxaemia and acidaemia, which may result in cerebral vasodilation, brain oedema formation and neuronal injury [34]. In encephalopathic COVID‐19 patients with acute respiratory distress syndrome, cerebral magnetic resonance imaging revealed bilateral frontotemporal hypoperfusion [35]. In addition, active smoking as another established risk factor in this study may favour oxidative stress, chronic inflammation and microvascular thrombosis, and was recently suggested to play a causal role for COVID‐19 severity [36].

Mental health disorders such as anxiety, depressed mood and PTSD were high in our cohort without significant improvement over time. Both disease‐specific mechanisms and restrictions in the individual's life during the pandemic with a recent decline of global mental health [37] may serve as an explanation. Whilst the latter could not be adjusted for, it was found that mechanical ventilation during acute COVID‐19 and pathological findings on the chest CT scan obtained at the 3‐month follow‐up were associated with anxiety or depression after 1 year. Neuropsychiatric symptoms including anxiety, depression and PTSDs are common in ICU survivors irrespective of COVID‐19 [38]. The identified risk factors in our study, which are associated with a more severe disease course, impose a higher likelihood of chronic complaints, which limit full recovery and trigger physical and mental illness.

Ten (12%) patients with a neurological disease at the 1‐year follow‐up which was not diagnosed prior to COVID‐19 were identified. It should be emphasized that most of the identified diseases were mild. Although a significant improvement in prevalence rates of neurological diseases could not be shown, individual patients recovered, especially those with CIP/CIM, underlining the importance of neuro‐rehabilitation in severe COVID‐19 patients. Similarly, positioning‐related peripheral nerve injury was alleviated in one patient [39]. There are insufficient data of other infectious diseases to be compared to long‐term neurological deficits as described in our study. This is also true for patients with influenza, where various neurological complications are described during the acute and post‐acute phase especially in children [40]; however, reports on long‐term neurological sequelae are lacking. In a recent large retrospective International Classification of Diseases tenth revision (ICD‐10) based study, higher rates of neurological and psychiatric diagnoses were described within 6 months after COVID‐19 compared to patients with influenza including intracranial haemorrhage, ischaemic stroke, parkinsonism, dementia or anxiety disorder [41]

A high prevalence of neurological signs was found in the detailed neurological evaluation (64% when including objective hyposmia), where improvement in individuals was as frequent as the observation of a new finding. Objective hyposmia was highly prevalent (51%) and comparable to 3‐month follow‐up (45%). Olfactory dysfunction may be a consequence of either upper respiratory epithelial cell injury including blocking of the olfactory cleft or neuronal cell damage leading to structural abnormalities of the olfactory bulb, primary olfactory cortex or secondary projection areas [42]. In this context, it is important to keep in mind that 22% of the general population are considered to have olfactory dysfunction [43], which is even higher when using objective measures compared to subjective measures. This may to some extent explain the discrepancy of objective hyposmia and perceived new hyposmia secondary to COVID‐19 (15%). The only factor that predicted objective hyposmia at the 1‐year follow‐up was advanced age. Independent of COVID‐19, olfactory function decreases with age [11]. Because odour identification (used by us) and odour discrimination declined to a lesser extent compared to odour thresholds, it was decided to use a uniform established cut‐off of 13 points. The absence of specific risk factors suggests that hyposmia may be a remnant of acute COVID‐19 like after other viral infections [44, 45, 46].

Our study has limitations. First, the design does not allow causality to be inferred between COVID‐19 and the reported neurological symptoms and diseases. Hence, the possibility of a chance association cannot be excluded. To overcome this shortcoming, pre‐existing neurological disorders were carefully evaluated and only neurological diseases that were not diagnosed before COVID‐19 are reported. Furthermore, longitudinal follow‐up data are provided. Secondly, due to the low patient numbers significant improvements may have been missed. Thirdly, a relatively high drop‐out rate was encountered at 12 months (44%); however, the patients were comparable in terms of disease severity and age, minimizing the risk of a substantial selection bias. Two patients died between hospital discharge and 12‐month follow‐up secondary to traumatic brain injury and acute myeloid leukaemia. Next, patients who died during the acute phase (19%) were not systematically assessed for neurological complications.

## CONCLUSIONS

Our study suggests a high prevalence of neurological and neuropsychiatric sequelae 1 year after COVID‐19 with 12% having a new onset neurological disease within 12 months post‐COVID. The most common symptom described was fatigue, independent of COVID‐19 disease severity. Our data underline the high global post‐acute disease burden calling for long‐term multidisciplinary management of these patients.

## CONFLICT OF INTEREST

KS reports grants from FWF Austrian Science Fund, grants from Michael J. Fox Foundation, grants from International Parkinson and Movement Disorder Society, personal fees from Teva, personal fees from UCB, personal fees from Lundbeck, personal fees from AOP Orphan Pharmaceuticals AG, personal fees from Abbvie, personal fees from Roche, personal fees from Grünenthal; all outside the submitted work. PM reports grants from TWF (Tyrolean Science Fund), grants from Medtronic, personal fees from Boston Scientific, all outside the submitted work. The other authors have nothing to disclose. All other authors declare no competing interests.

## AUTHOR CONTRIBUTION

Verena Rass: Conceptualization (lead); data curation (equal); formal analysis (lead); investigation (equal); methodology (equal); project administration (lead); visualization (lead); writing—original draft (lead). Ronny Beer: Data curation (equal); supervision (equal); writing—review and editing (equal). Alois Josef Schiefecker: Data curation (equal); writing—review and editing (equal). Anna Lindner: Data curation (equal); writing—review and editing (equal). Mario Kofler: Data curation (equal); writing—review and editing (equal). Bogdan Ianosi: Formal analysis (equal); visualization (equal); writing—review and editing (equal). Philipp Mahlknecht: Data curation (equal); writing—review and editing (equal). Beatrice Heim: Data curation (equal); writing—review and editing (equal). Marina Peball: Data curation (equal); writing—review and editing (equal). Federico Carbone: Data curation (equal); writing—review and editing (equal). Victoria Limmert: Project administration (equal); writing—review and editing (equal). Philipp Kindl: Data curation (equal); writing—review and editing (equal). Lauma Putnina: Data curation (equal); writing—review and editing (equal). Elena Fava: Data curation (equal); writing—review and editing (equal). Sabina Sahanic: Data curation (equal); writing—review and editing (equal). Thomas Sonnweber: Conceptualization (equal); data curation (equal); writing—review and editing (equal). Wolfgang Loescher: Data curation (equal); writing—review and editing (equal). Julia Wanschitz: Data curation (equal); writing—review and editing (equal). Laura Zamarian: Data curation (equal); writing—review and editing (equal). Atbin Djamshidian: Data curation (equal); writing—review and editing (equal). Ivan Tancevski: Conceptualization (equal); data curation (equal); writing—review and editing (equal). Guenter Weiss: Supervision (equal); writing—review and editing (equal). Rosa Bellmann: Supervision (equal); writing—review and editing (equal). Stefan Kiechl: Conceptualization (equal); supervision (equal); writing—review and editing (equal). Klaus Seppi: Data curation (equal); writing—review and editing (equal). Judith Loeffler‐Ragg: Conceptualization (equal); supervision (equal); writing—review and editing (equal). Bettina Pfausler: Data curation (equal); supervision (equal); writing—review and editing (equal). Raimund Helbok: Conceptualization (lead); data curation (equal); investigation (equal); methodology (equal); project administration (lead); supervision (lead); writing—original draft (equal); writing—review and editing (equal).

## Data Availability

The data that support the findings of this study are available from the corresponding author upon reasonable request and fulfilling data sharing regulations approved by the local ethics committee.
