# Supplementary material for: Neurological outcomes 1 year after COVID‐19 diagnosis: A prospective longitudinal cohort study
Source: Eur J Neurol. 2022 Mar 23;29(6):1685–96. doi: 10.1111/ene.15307 (PMC9111823; doi:10.1111/ene.15307)
Supplement: Supplementary file 1 [file ENE-29-1685-s001.docx]

| **Supplemental Table 1. Neurologic diseases at 1-year follow-up not diagnosed prior to COVID-19 across severity grades.** | | | | | |
| --- | --- | --- | --- | --- | --- |
|  | All  n=81 | Severe disease requiring ICU admission  n=20 (25%) | Moderate severity  (Hospitalization- non-ICU) n=35 (43%) | Mild severity (Outpatient)  n=26 (32%) | P-value* |
| Any neurological disease not diagnosed prior to COVID-19 | 9 (11) | 5 (25) | 3 (9) | 1 (4) | 0.063 |
| Neuropathy/myopathy | 7 (9) | 4 (20) | 3 (9) | 1 (4) | 0.180 |
| CIP/CIM | 1 (1) | 1 (5) | 0 (0) | 0 (0) | 0.214 |
| Symmetric axonal distal neuropathy | 3 (4) | 1 (5) | 2 (6) | 1 (4) | 0.946 |
| Small fibre polyneuropathy | 1 (1) | 1 (5) | 0 (0) | 0 (0) | 0.214 |
| Compression neuropathy | 3 (4) | 1 (5) | 1 (3) | 1 (4) | 0.920 |
| Guillain‐Barré syndrome | 0 (0) | 0 (0) | 0 (0) | 0 (0) | … |
| Parkinsonism | 2 (2) | 1 (5) | 1 (3) | 0 (0) | 0.546 |
| Cerebellar ataxia | 0 (0) | 0 (0) | 0 (0) | 0 (0) | … |
| Mild encephalopathy | 1 (1) | 1 (5) | 0 (0) | 0 (0) | 0.214 |
| Ischemic stroke | 0 (0) | 0 (0) | 0 (0) | 0 (0) | … |
| Hemorrhagic stroke | 0 (0) | 0 (0) | 0 (0) | 0 (0) | … |
| Orthostatic hypotension | 0 (0) | 0 (0) | 0 (0) | 0 (0) | … |
| Seizures | 0 (0) | 0 (0) | 0 (0) | 0 (0) | … |
| Myelopathy | 0 (0) | 0 (0) | 0 (0) | 0 (0) | … |
| Data are given in counts (%).  *The chi-square test was used to assess for differences across severity grades (severe, moderate, mild). A P-value <0.05 signifies a significant different data distribution across severity groups.  Abbreviations: CIP/CIM - Critical illness poly-neuropathy and myopathy. | | | | | |

| **Supplemental Table 2: Neurological signs and symptoms one year after COVID-19 diagnosis across severity grades.** | | | | | |
| --- | --- | --- | --- | --- | --- |
|  | All  n=81 | Severe disease requiring ICU admission  n=20 (25%) | Moderate severity (Hospitalization, non-ICU)  n=35 (43%) | Mild severity (Outpatient)  n=26 (32%) | p value* |
| Any neurological sign or symptom | 52 (64) | 16 (80) | 26 (74) | 10 (39) | 0.004 |
| Hyposmia/anosmia,  SS-16 <13 | 41 (51) | 13 (65) | 18 (51) | 10 (40) | 0.249 |
| Neck stiffness | 0 (0) | 0 (0) | 0 (0) | 0 (0) | … |
| Decreased consciousness | 0 (0) | 0 (0) | 0 (0) | 0 (0) | … |
| Dysarthria | 0 (0) | 0 (0) | 0 (0) | 0 (0) | … |
| Aphasia | 0 (0) | 0 (0) | 0 (0) | 0 (0) | … |
| Positive frontal release signs | 8 (10) | 0 (0) | 8 (23) | 0 (0) | 0.003 |
| Anisocoria | 0 (0) | 0 (0) | 0 (0) | 0 (0) | … |
| Oculomotor nerve palsy | 0 (0) | 0 (0) | 0 (0) | 0 (0) | … |
| Facial palsy | 0 (0) | 0 (0) | 0 (0) | 0 (0) | … |
| Dysphagia | 0 (0) | 0 (0) | 0 (0) | 0 (0) | … |
| Bradykinesia | 5 (6) | 2 (10) | 3 (9) | 0 (0) | 0.277 |
| Dystonia | 0 (0) | 0 (0) | 0 (0) | 0 (0) | … |
| Chorea | 0 (0) | 0 (0) | 0 (0) | 0 (0) | … |
| Myoclonus/jerks | 0 (0) | 0 (0) | 0 (0) | 0 (0) | … |
| Asterixis | 0 (0) | 0 (0) | 0 (0) | 0 (0) | … |
| Dysmetria | 2 (2) | 0 (0) | 2 (6) | 0 (0) | 0.260 |
| Tremors | 2 (2) | 0 (0) | 2 (6) | 0 (0) | 0.260 |
| Abnormal muscle tone | 5 (6) | 2 (10) | 3 (9) | 0 (0) | 0.277 |
| Rigidity | 5 (6) | 2 (10) | 3 (8) | 0 (0) | 0.277 |
| Spasticity | 0 (0) | 0 (0) | 0 (0) | 0 (0) | … |
| Decreased muscle tone | 0 (0) | 0 (0) | 0 (0) | 0 (0) | … |
| Muscle atrophy | 3 (4) | 3 (15) | 0 (0) | 0 (0) | 0.009 |
| Decreased/disturbed sensation | 14 (17) | 7 (35) | 5 (14) | 2 (8) | 0.043 |
| Abnormal reflex status | 16 (20) | 6 (30) | 8 (23) | 2 (8) | 0.141 |
| Paresis | 4 (5) | 3 (15) | 1 (3) | 0 (0) | 0.050 |
| Babinski sign | 1 (1) | 0 (0) | 1 (3) | 0 (0) | 0.514 |
| Gait abnormality | 6 (7) | 3 (15) | 3 (9) | 0 (0) | 0.147 |
| Data are given as count (%).  *The chi-square test was used to assess for differences across severity grades (severe, moderate, mild). A P-value <0.05 signifies a significant different data distribution across severity groups.  Abbreviations: ICU - intensive care unit; SS‐16 - 16‐item Sniffin’ Sticks test. | | | | | |

| **Supplemental Table 3. Mental health, cognition, and functional outcome three months and one year after COVID-19 diagnosis.** | | | | | |
| --- | --- | --- | --- | --- | --- |
|  | 1-year FU  n=81 | Severe disease requiring ICU admission  n=20 (25%) | Moderate severity (Hospitalization, non-ICU)  n=35 (43%) | Mild severity (=Outpatient)  n=26 (32%) | p-value* |
| **Mental health** | | | | | |
| Post-traumatic stress disorder; PCL 5 >32 | 5 (10) | 2 (15) | 2 (10) | 1 (6) | 0.714 |
| Depression (HADS-D) | 3 (6) | 1 (7) | 0 (0) | 2 (13) | 0.256 |
| Depression (HADS-D) >7 | 1 (2) | 0 (0) | 0 (0) | 1 (6) | … |
| Depression (HADS-D) >10 | 2 (4) | 1 (7) | 0 (0) | 1 (6) | … |
| Anxiety (HADS-A) | 15 (29) | 5 (36) | 8 (36) | 2 (13) | 0.222 |
| Anxiety (HADS-A) >7 | 9 (17) | 3 (21) | 5 (23) | 1 (6) | … |
| Anxiety (HADS-A) >10 | 6 (12) | 2 (14) | 3 (14) | 1 (6) | … |
| **Fatigue measures** | | | | | |
| FAS | 22 (18-26) | 24 (18-30) | 21 (18-26) | 19 (17-25) | 0.260 |
| FAS >21 | 34 (50) | 10 (67) | 14 (47) | 10 (44) | 0.334 |
| FSS | 3 (2-5) | 3 (2-5) | 3 (2-5) | 3 (2-4) | 0.559 |
| FSS ≥5 | 11 (19) | 4 (25) | 4 (17) | 3 (17) | 0.791 |
| **Cognition** | | | | | |
| MoCA (<26) | 14 (18) | 4 (24) | 10 (30) | 0 (0) | 0.010 |
| MoCA | 28 (26-29) | 27 (26-28) | 26 (25-29) | 29 (27-30) | 0.002 |
| **Functional outcome** | | | | | |
| GOSE | 8 (7-8) | 7 (7-8) | 8 (7-8) | 8 (7-8) | 0.463 |
| mRS | 0 (0-1) | 1 (0-1) | 0 (0-1) | 0 (0-1) | 0.884 |
| Data are given in median (interquartile range) and counts (%).  *Chi-square or Kruskal-Wallis tests were used to assess for differences across severity grades.  Abbreviations: PCL-5 - Checklist for posttraumatic stress disorder, HADS-D - Hospital Anxiety and Depression Scale, MoCA - Montreal - Cognitive Assessment; FAS - fatigue assessment scale; FSS - fatigue severity scale; GOSE - Glasgow Outcome Scale Extended; mRS – modified Rankin Scale score.  Anxiety and depression (HADS-D) were scored as slightly increased when >7 and increased when >10. | | | | | |
